# Aphid adaptation to cucurbits: sugars, cucurbitacin and phloem structure in resistant and susceptible melons

**DOI:** 10.1186/s12870-023-04248-1

**Published:** 2023-05-05

**Authors:** Pierre Sadon, Marie-Noëlle Corre, Raphael Lugan, Nathalie Boissot

**Affiliations:** 1 Génétique et Amélioration des Fruits et Légumes, National Institute for Agriculture, Food and Environment, INRAE, Domaine St-Maurice, 84143 Montfavet, Cedex France; 2 Plantes et Systèmes de cultures Horticoles, National Institute for Agriculture, Food and Environment, INRAE, Domaine St Paul, 84914 Avignon, Cedex France

**Keywords:** *NLR*, Plant resistance, Specialized metabolites, Primary metabolites, *Vat* melon

## Abstract

**Background:**

*Aphis gossypii,* a strictly phloemophagaous aphid*,* colonize hundreds of plant families, and a group of clones formed a cucurbit-specialised host-race. Cucurbits are unique in having evolved a specific extra-fascicular phloem (EFP), which carries defence-related metabolites such as cucurbitacin, whereas the fascicular phloem (FP) is common to all higher plants and carries primary metabolites, such as raffinose-family oligosaccharides (RFOs). Both cucurbitacins (in the EFP) and galactinol (in the FP) have been suggested to be toxic to aphids. We investigated these hypotheses in cucurbit-specialized *A. gossypii* fed on melon plants with or without aphid-resistance conferred by the NLR gene *Vat*. We selected a plant-aphid system with (i) *Vat*-mediated resistance not triggered, (ii) *Vat*-mediated resistance triggered by an aphid clone adapted to the presence of *Vat* resistant alleles and (iii) *Vat*-mediated resistance triggered by a non-adapted aphid clone.

**Results:**

We quantified cucurbitacin B, its glycosylated derivative, and sugars, in melon plants and aphids that fed on. The level of cucurbitacin in plants was unrelated to both aphid infestation and aphid resistance. Galactinol was present at higher quantities in plants when *Vat*-mediated resistance was triggered, but its presence did not correlate with aphid performance. Finally, we showed that cucurbit-specialized *A. gossypii* fed from the FP but could also occasionally access the EFP without sustainably feeding from it. However, the clone not adapted to *Vat*-mediated resistance were less able to access the FP when the *Vat* resistance was triggered.

**Conclusion:**

We concluded that galactinol accumulation in resistant plants does not affect aphids, but may play a role in aphid adaptation to fasting and that Cucurbitacin *in planta* is not a real threat to *Aphis gossypii.* Moreover, the specific phloem of Cucurbits is involved neither in *A. gossypii* cucurbit specialisation nor in adaptation to *Vat*-dependent resistance.

**Supplementary Information:**

The online version contains supplementary material available at 10.1186/s12870-023-04248-1.

## Background

Vascular plants colonized land about 400 million years ago and 300 million years later aphids evolved specialized mouthparts called stylets, which could penetrate plant tissues to establish an intricate relationship with this vascular system that is their feeding source [27]. About 4000 aphid species are known, including the melon-cotton aphid, *Aphis gossypii*, which diverged about 5 million years ago (http://timetree.org). *A. gossypii* is an extremely polyphagous pest with populations structured into host-specific races feeding and reproducing on different crops [2, 3, 6], including a specialist aphid for cucurbitaceous species [24].

Cucurbits, which diverged 60 million years ago (http://timetree.org) earlier than *A. gossypii*, exhibit an unusual vascular system which is split into two physically and functionally distinct phloems [40]. The fascicular phloem (FP), composed of bundles of sieve tubes and companion cells on both sides of the xylem (bicollateral), is mainly involved in the transport of photoassimilates. The extrafascicular phloem (EFP) is composed of an array of phloem elements located outside the bundles and is mainly involved in the transport of various metabolites, including defence-related compounds [40]. The FP is common to higher plants whereas the EFP is only found in the Cucurbitaceae family [34]. Different EFP configurations exist in Cucurbitaceae, depending on the species and its phylogeny: *Alsomitra*, for instance, displays only collateral (rather than bicollateral) bundles, while *Cucurbita* has the most complex phloem type with a highly developed extrafascicular phloem, featuring all types of extrafascicular elements [34]. Knowledge on the interaction between aphids and the cucurbit vascular system is quite sparse. Electrical-Penetration Graph (EPG) recordings brought direct evidence that aphids feed from the melon vascular system [7, 22], but no EPG waveform has been able to discriminate between FP or EFP feeding aphids thus far. Despite the suggestion that the EFP might contain a dietary composition more suited to aphids due to its relatively low sugar/amino acid content [14, 23], the comparison of sugar profiles from *Myzus persicae* stylet exudates with sieve exudates from FP and EFP indicated that *M. persicae* fed exclusively from FP on *Cucurbita pepo* [20]. Moreover, the location of stylet track endings, as shown using microscopy in *Cucurbita pepo*, suggested that *A. gossypii* fed on FP, and since feeding on artificial diets supplemented with EFP exudates had a negative impact on their growth rate after 72 h, aphids have been suggested to show EFP avoidance behaviour [20].

Melon is of particular interest for the plant-aphid interaction given that it is the only cucurbit for which a high level of resistance to *A. gossypii* has been detected. This resistance is mediated by the Nucleotide-binding domain—Leucine-rich Repeat (NLR) gene named *Vat* [12]. EPG profiles in tissues showed that aphid feeding behaviour is impaired on *Vat*-melon plants, *i.e.* plants carrying a resistant allele in the *Vat* gene cluster [9]: aphids reach the phloem less often and feed for a shorter time on *Vat-*plants compared to susceptible plants [26, 37]. Callose and lignin deposition in the walls of cells adjacent to the stylet path, and putatively also on the sieve-plates in the phloem, occurs faster in *Vat*-melon plants [17, 26, 37]. Such phloem obstruction was also observed after mechanical wounding in both FP and EFP, but later and to a lesser extent for the EFP [10, 38, 40]. Yet, some clones are adapted to *Vat*-mediated resistance: they can survive, reproduce and cause injury to *Vat*-melon plants [3]. Insight into the spectrum of resistance conferred by *Vat* suggests that *A. gossypii* adaptation to *Vat*-mediated resistance occurs by two different mechanisms: (i) following the effector triggered immunity model for NLR resistance proposed by Jones and Dangl [11] whereby an alteration in the avirulence salivary component results in loss of recognition by the VAT NLR protein, preventing the triggering of resistance and (ii) in the absence of alteration to the avirulence salivary component, aphids cope with *Vat* resistance through unknown mechanisms [2, 3].

**Fig. 1:**
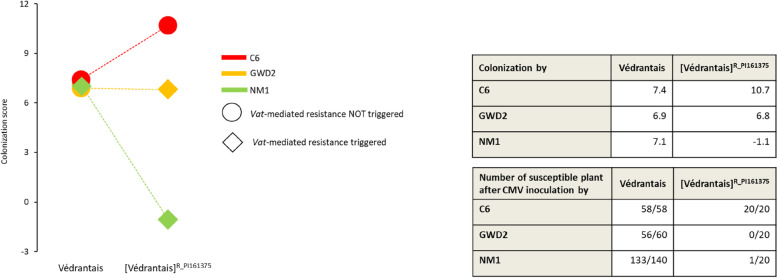
Interactions between three aphid clones and two isogenic melon lines Védrantais and [Védrantais]^R_PI161375^. ‘Colonization’ by aphids is a quantitative trait inferred from the number of nymphs and adults seven days after a controlled infestation. Vat-mediated resistance is triggered when plants are resistant to virus after aphid-inoculation with Cucumber mosaic virus, and NOT triggered when plants are susceptible. Data in both tables are from Additional file 1 from [3]

The timeline of aphid and cucurbit evolutionary history is consistent with the putative co-evolution of the cucurbit-specialised *A. gossypii* host-race feeding from the EFP and adapting to the defence-related compounds present. In the present study, we investigated whether the cucurbit-specialised *A. gossypii* host-race and the *Vat*-adapted clones could use the EFP as an alternative feeding source on susceptible or resistant melon plants. We selected a small set of metabolites typically found in FP and EFP: for FP we chose raffinose-family oligosaccharides (RFOs) [31, 40] and trehalose, with galactinol being potentially toxic to aphids [4, 5]. For EFP, we selected cucurbitacin as being potentially toxic to aphids [39, 42]. We developed a GC–MS method to analyse RFOs and trehalose in plants and aphids, and an LC–MS method to analyse cucurbitacin B (Cb) [18, 40]. We looked at the abundance and variability of these metabolites in a variety of melon lines. We employed two sources of *Vat* resistance: one from the Korean melon line PI 161,375 and the other from the Zimbabwean line PI 482,398, as well as two pairs of lines which differ for the presence of the *Vat* allele derived from the resistant lines: in the first case, the *Vat* gene from PI 161,375 was introduced by transgenesis into a Védrantais background, and in the second, the *Vat* resistance from PI 482,398 was introduced into a Charentais T background by backcrossing. We named these two pairs of lines Védrantais/[Védrantais]^R_PI161375^ and Charentais T/[Charentais T]^R_PI482398^. The metabolic markers were screened in three plant-aphid interaction systems using the pair of isogenic melon lines Védrantais/[Védrantais]^R_PI161375^ and three aphid clones: NM1, C6 and GWD2 with the following response on [Védrantais]^R_PI161375^: (i) NM1 triggers *Vat-*mediated resistance and is not adapted to that resistance, (ii) GWD2 triggers *Vat-*mediated resistance and is adapted to that resistance and (iii), C6 which does not trigger the *Vat-*mediated resistance (Fig. 1) [3].

## Results

### Abundance and variability of phloem-associated metabolites in six melon genotypes

**Fig. 2:**
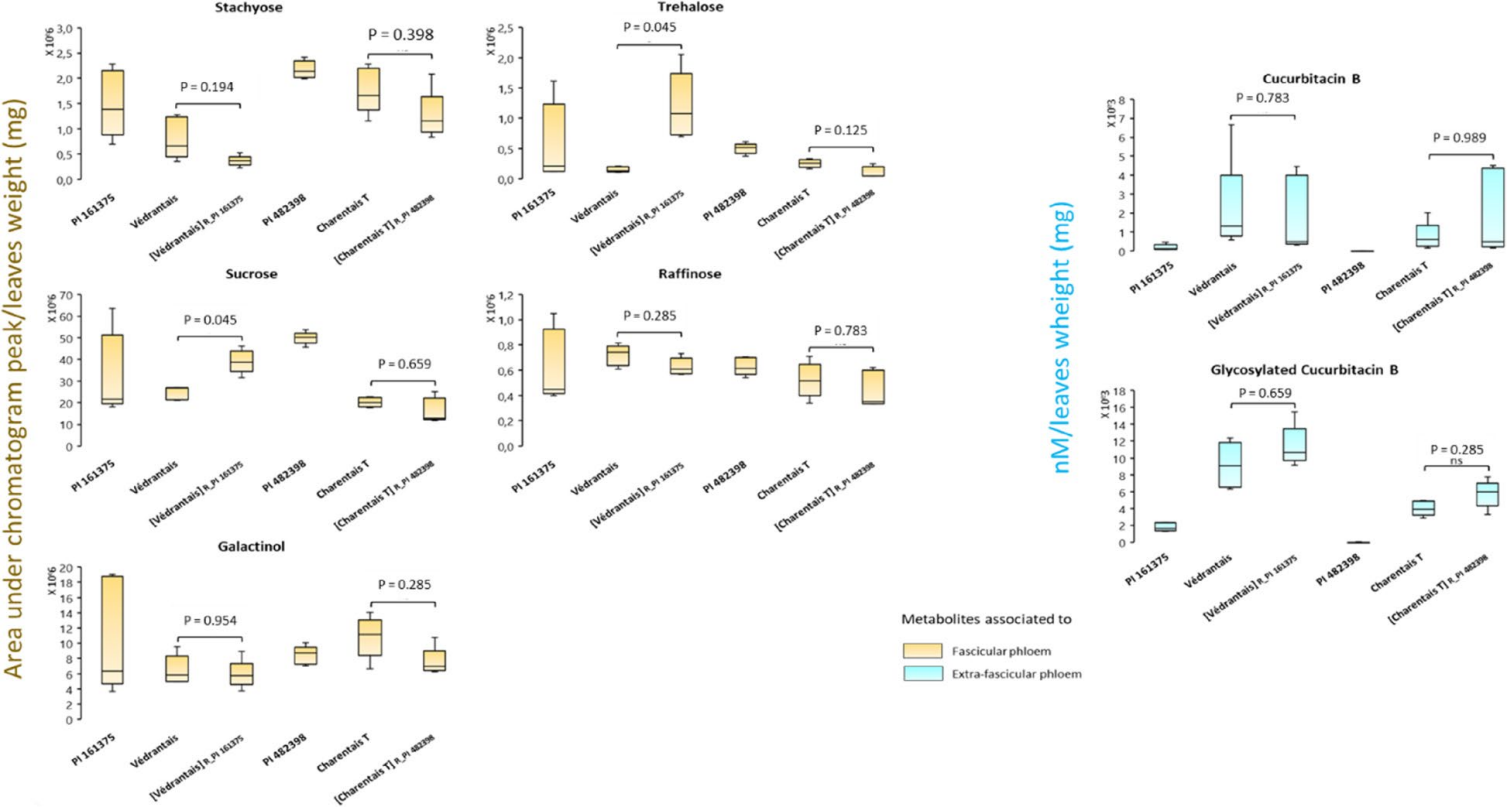
Quantification of seven phloem-associated metabolites in six melon lines. Metabolites associated with the melon fascicular phloem were analysed by GC–MS (displayed in orange) and quantified as the area under the chromatogram peak (no units) corrected by leaf sample weight (g). Metabolites associated with the melon extra-fascicular phloem were analysed by LC–MS (displayed in light blue) and expressed as nM.mg^−1^. Five melon plants were analysed four each melon genotype. PI 161375 and PI 482398 carry different *Vat* resistance alleles, Védrantais and Charentais T are susceptible to aphids. Their isogenic and near-isogenic lines with resistance donors are noted [Védrantais]^R_PI 161375^ and [Charentais]^R_PI 482398^. Statistical comparisons were made between the Védrantais isogenic lines and between the Charentais T near-isogenic lines using a non- parametric bilateral Kruskal–Wallis test

We quantified seven phloem-associated metabolites in the parental lines and the pairs of isogenic and near-isogenic lines (Fig. 2 and Sup_Tab. 1). The five FP-associated metabolites were detected in all lines with significant differences (Kruskal and Wallis tests) for stachyose (*p* = 0.001), sucrose (*p* = 0.001), trehalose (*p* = 0.001) and both forms of cucurbitacin (*p* < 0.002). The concentrations of sucrose and trehalose showed significant differences between [Védrantais]^R_PI161375^ and Védrantais but not significant between [Charentais T]^R_PI482398^ and Charentais T. This contrast was unexpected as Védrantais and its transgenic line [Védrantais]^R_PI161375^ differ only for the *Vat* transgene while the near -isogenic lines Charentais T/[Charentais T]^R_PI482398^ differ for about 2% of their genome. Remarkably, both resistance donor lines, PI 161,375 and PI 482,398, contained low amounts of Cb and Cb-Glc, when detectable, which meant they were unsuitable for the investigation into putative ingestion of EFP-associated metabolites by aphids. Pairs of Védrantais and Charentais T lines however, contained higher amounts of Cb and Cb-Glc than the resistant parental lines.

**Fig. 3:**
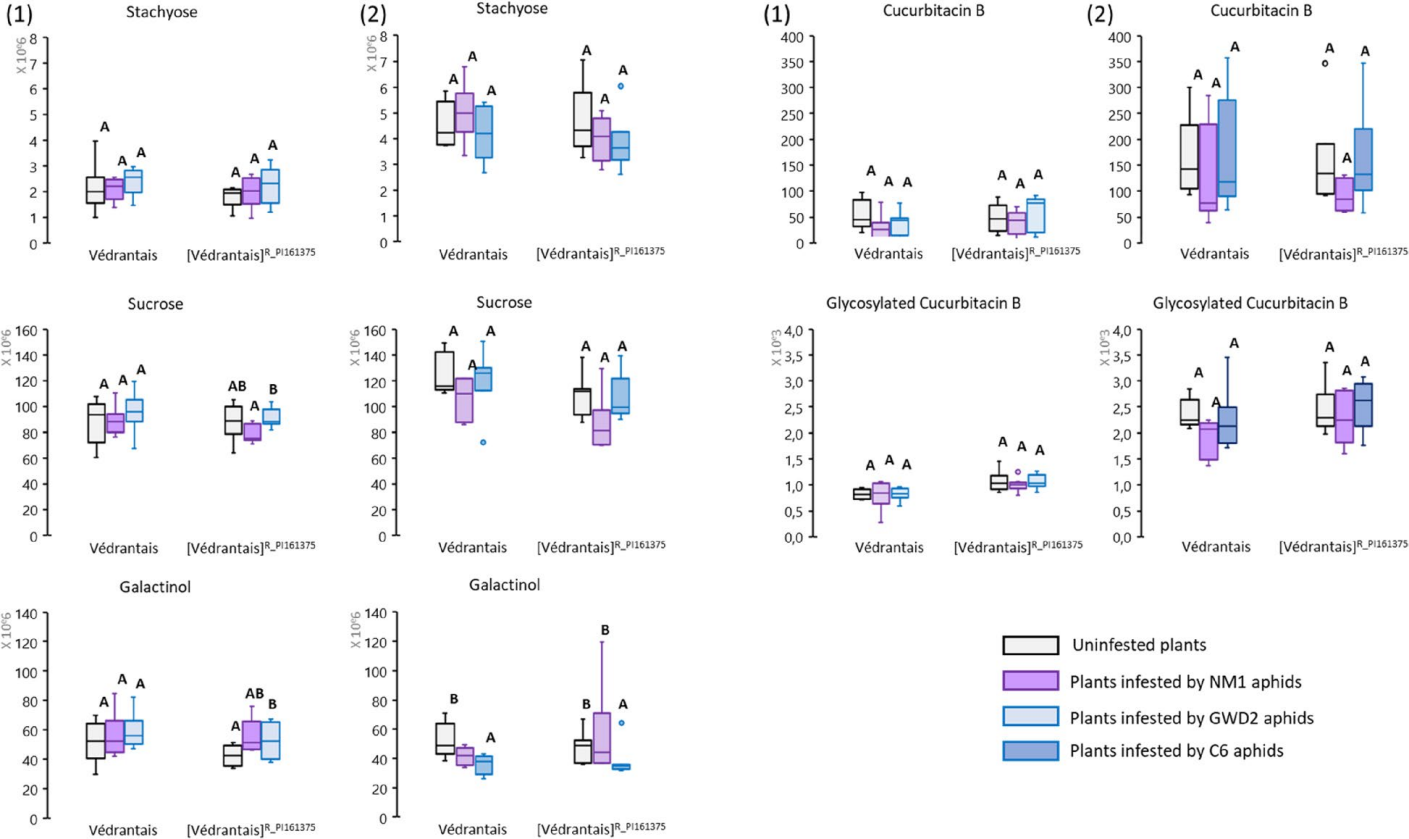
Quantification of five phloem-associated metabolites in healthy versus aphid-infested melon plants. Védrantais and its isogenic [Védrantais]^R_PI 161375^ plants were infested for 17 h by NM1 aphid clone -triggering the *Vat*-mediated resistance- in both experiments, and by either GWD2 aphids -triggering too the *Vat*-mediated resistance- in experiment (1) or C6 aphids -non-triggering the *Vat*-mediated resistance in experiment (2). Stachyose, sucrose and galactinol were analysed by GC–MS in melon leaves and expressed as peak area (no units) corrected by leaf sample weight (g). Cucurbitacin B and Glycosylated Cucurbitacin B were analysed by LC–MS and expressed as nMole.mg^−1^. The number of observations per melon genotype was between 6 and 9. Statistical comparisons were made between infestation conditions for a melon genotype (letter codes indicate significant differences) using a non- parametric bilateral Kruskal–Wallis test at alpha = 0.05

Using Védrantais/[Védrantais]^R_PI161375^ we investigated whether the infestation of plants by aphids induced modification of sugar and cucurbitacin content compared to healthy plants (Fig. 3 and Sup_Tab. 2).

Levels of stachyose and sucrose in aphid-infested melon plants did not significantly differ from those of healthy plants, regardless of the aphid clone and the melon genotype. In contrast, galactinol levels were different in infested compared to healthy melon plants. Interestingly, galactinol levels were higher in GWD2-infested [Védrantais]^R_PI161375^ melon plants than in healthy [Védrantais]^R_PI161375^ while galactinol levels were lower in C6-infested [Védrantais]^R_PI161375^ than in healthy [Védrantais]^R_PI161375^. No significant differences were observed in the levels of cucurbitacin B and its glycoside in any of the plant-clone combinations compared to healthy plants (Fig. 3).

### Identification of phloem-associated metabolites accumulated by aphids

**Fig. 4:**
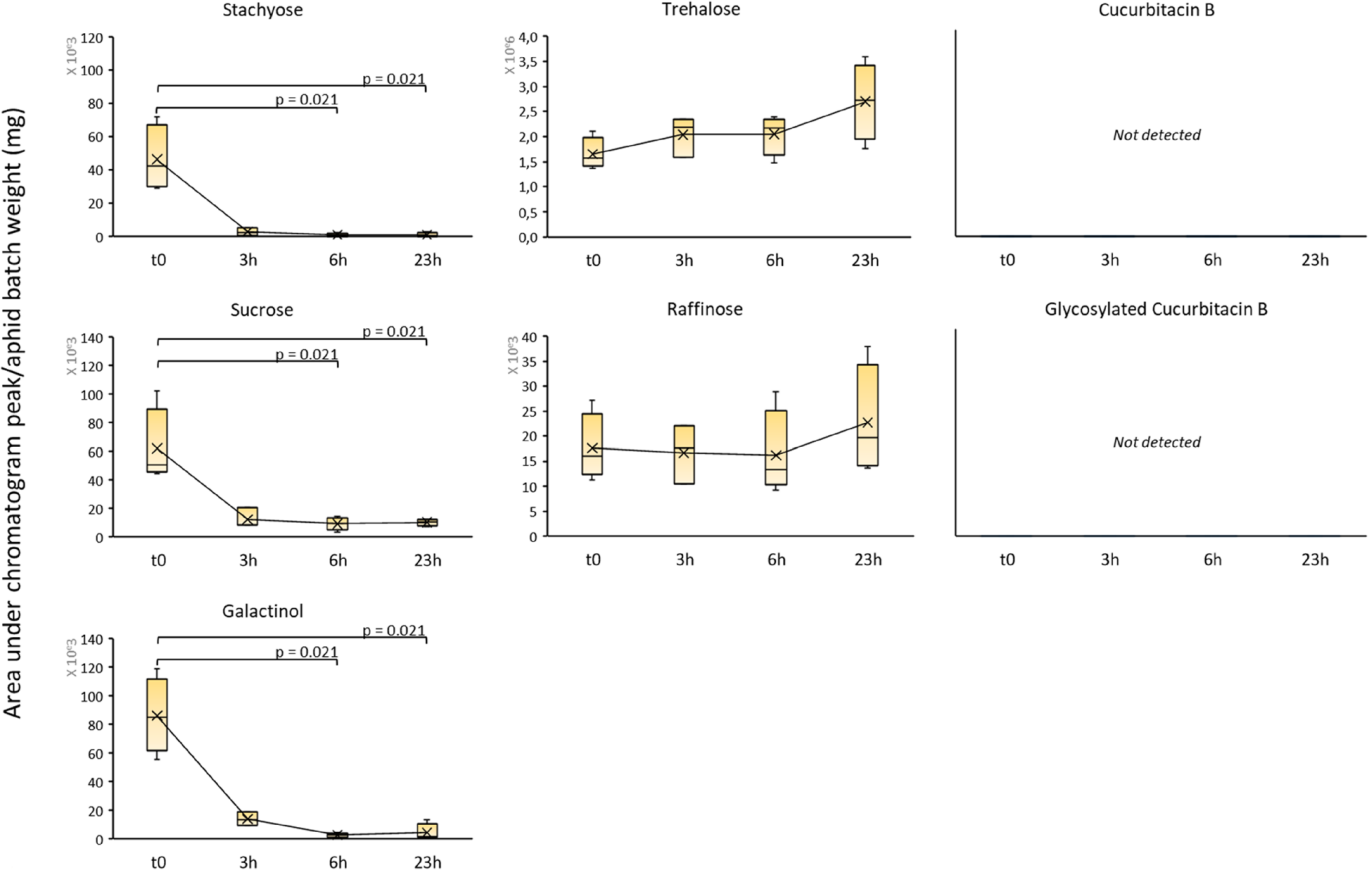
Seven melon-associated metabolites in aphids during fasting. C6 adult aphids were reared on Védrantais melon plants, then fasted by batches of 20 to 50 individuals for 3 h, 6 h and 23 h. t0 corresponds to the aphids sampled at rearing (before fasting). For each time point, four aphid batches were sampled, except for the “3 h” point where three aphid batches were sampled. Stachyose, sucrose and galactinol were analysed by GC–MS in aphid batches and expressed as peak area (no units) corrected by the aphid batch weight (mg). Cucurbitacin B and Glycosylated Cucurbitacin B were not detected by LC–MS. The metabolite contents in aphid batches at t = 3 h, 6 h and 23 h were compared to t0 using a unilateral non-parametric Kruskal–Wallis test corresponding to alpha = 0.05 (i.e. alpha = 0.025). Only significant differences are marked in the figure

To ensure that most of the melon phloem-associated metabolites found in aphids were accumulated during the experiment, we purged aphids after mass rearing, either by allowing them to feed on a plant free of the cucurbit marker metabolites, *i.e.* okra, or by fasting aphids in empty petri dishes. As expected, in two-week-old okra leaves, neither stachyose nor Cb were detected (Fig. S1 and Sup_Tab. 3). Rearing aphids on okra was unsuccessful however: after two weeks, the aphids had poorly colonized the plants and showed noticeable weight loss and had laid very few eggs. These dramatic effects on the biological performance of aphids ultimately led to the disqualification of okra as a system for purging aphids. The fasting alternative was tested for 3 h, 6 h and 23 h on 3 to 4 batches of 20 to 50 adults of the C6 clone, previously reared on Védrantais melon plants. The metabolite quantities in aphid batches are shown in (Fig. 4 and Sup_Tab. 4): average amounts of stachyose, sucrose and galactinol decreased by 94, 80 and 84% respectively after 3 h of fasting and the decrease was significant after 6 h. Thereafter, and until the end of the fasting period, the quantity of these three sugars remained stable and similar to the quantities recorded at t = 3 h. In contrast, average amounts of raffinose and trehalose remained stable during the entire fasting period. Notably, neither Cb nor Cb-Glc were detected in aphid batches. Therefore, between 3–6 h fasting appeared to be efficient in purging stachyose, sucrose and galactinol from aphids.

### Search for aphid-accumulated phloem metabolite markers

After 4-h of fasting, aphids were fed on either Védrantais or [Védrantais]^R_PI161375^ for 17 h. Metabolites were not available in same quantities for aphids according to the melon plant they fed on. To compare quantities of metabolites collected by each aphid batch, we considered the ratio [metabolite quantity in an aphid batch]/[metabolite quantity in the leaf on which they fed].

Each experiment focused on a *Vat*-adapted clone: GWD2 which triggers *Vat-*mediated resistance in [Védrantais]^R_PI161375^, and C6 which does not. In both experiments, NM1 was used as a reference for triggering of *Vat-*mediated resistance in [Védrantais]^R_PI161375^.

**Fig. 5:**
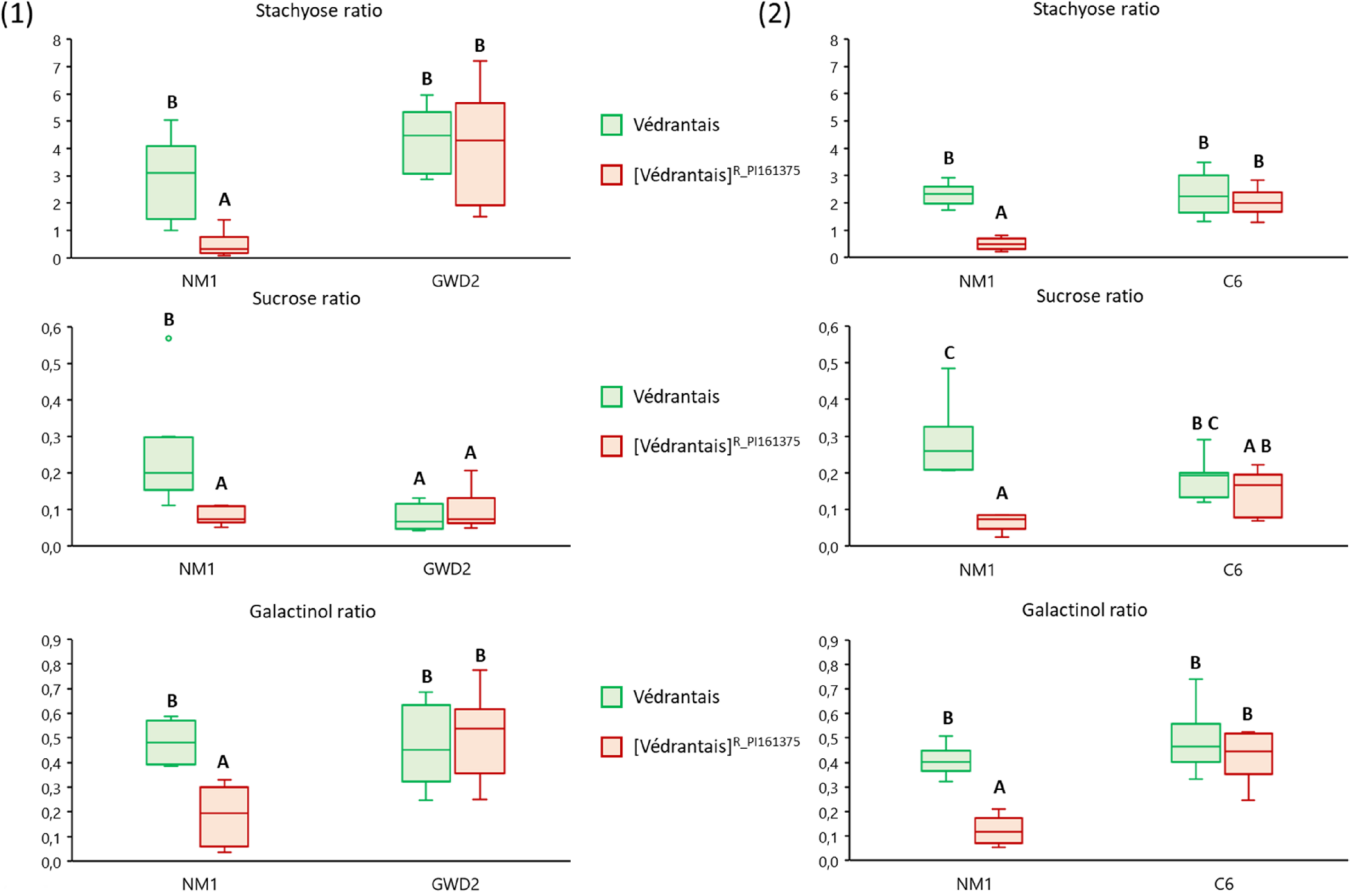
Stachyose, sucrose and galactinol in three aphid clones fed on Vat/non-Vat melons*.* Aphid clones were fed on Védrantais and its isogenic [Védrantais]^R_PI161375^ melon lines for 17 h. NM1 aphids -triggering the *Vat*-mediated resistance- were used in both experiments, with either GWD2 aphids -triggering the resistance in [Védrantais]^R_PI161375^- in experiment (1), or C6 aphids -non-triggering the resistance in [Védrantais]^R_PI161375^- in experiment (2). Results are ratios of [metabolite quantity in an aphid batch]/[metabolite quantity in the leaf on which they fed]. The number of observations is between 5 and 9. Statistical comparisons were made between conditions within each experiment for each metabolite (Letter codes indicate significant differences between all conditions within an experiment at alpha = 0.05 according to Kruskal–Wallis tests)

The three RFO related metabolites, stachyose, sucrose and galactinol, were detected in all aphid batches feeding on Védrantais and [Védrantais]^R_PI161375^ (Fig. 5 and Sup_Tab. 5; Fig. S2 and Sup_Tab. 6).

NM1 accumulated significantly less sugar on [Védrantais]^R_PI161375^ than on Védrantais (on average 6.2, 3.1 and 2.6 times less for stachyose, sucrose and galactinol respectively in the first experiment and 4.5, 4.2 and 3.4 times less in the second). On the contrary, both *Vat*-adapted clones, GWD2 in experiment 1 and C6 in experiment 2, accumulated similar amounts of sugar on [Védrantais]^R_PI161375^ and Védrantais.

Remarkably, no Cb-Glc was detected in the aphids from either experiment. In NM1 and C6, no Cb was detected after feeding on Védrantais or [Védrantais]^R_PI161375^, therefore, NM1 and C6 did not accumulate Cb after fasting, regardless of the plant genotype. In GWD2, Cb was detected only in 2/9 batches which fed on [Védrantais]^R_PI161375^ and only in very small amounts (Sup_Tab. 7). Therefore, few GWD2 aphids putatively accumulated Cb after feeding on [Védrantais]^R_PI161375^ or, alternatively, were not fully purged after mass rearing. This second hypothesis was supported by the discovery of very small amounts of Cb in 8/15 batches of fasted NM1 and GWD2 (Sup_Tab. 7).

**Fig. 6:**
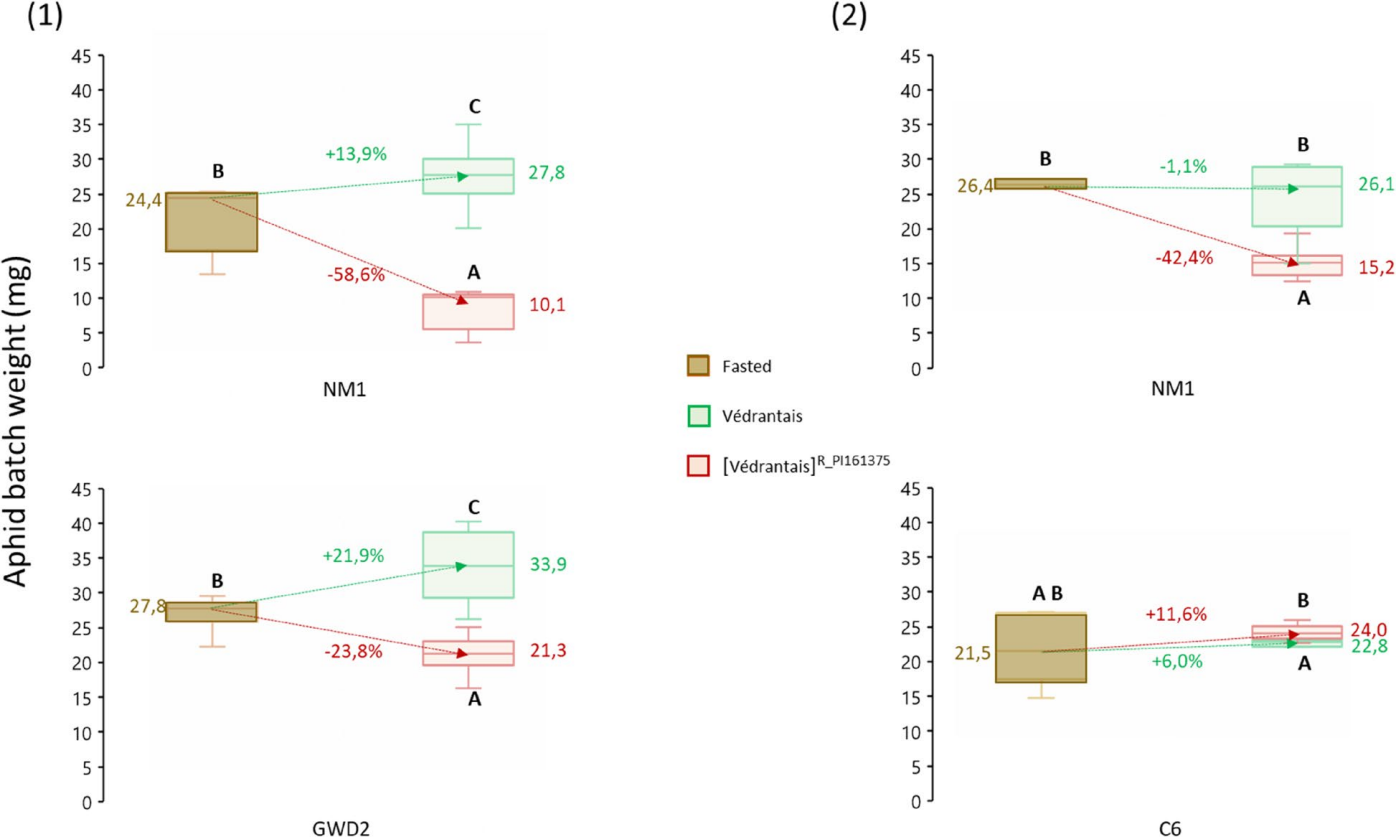
Weight (mg) of three aphid clones fasted and fed on Vat/non-Vat melons. Aphids were fasted for 4 h and then fed on Védrantais and its isogenic [Védrantais]^R_PI161375^ melon lines for 17 h. NM1 aphids -triggering the Vat-mediated resistance- were used in both experiments, with either GWD2 aphids -triggering the resistance in [Védrantais]^R_PI161375^- in experiment (1), or C6 aphids -non-triggering the resistance in [Védrantais]^R_PI161375^- in experiment (2). Arrows indicate the median loss/gain of weight of fasting aphid batches versus fed aphid batches. Between 3 and 6 aphid batches were analysed after fasting and between 7 and 9 after feeding. Statistical comparisons were made between conditions within each experiment (Letter codes indicate significant differences between fasted batches and fed batches at alpha = 0.05 according to Kruskal–Wallis tests)

In addition, we recorded the weight of aphid batches after a 4 h-fasting period as well as for each experimental condition following a 17 h-feeding period (Fig. 6 and Sup_Tab. 8).

After feeding on [Védrantais]^R_PI161375^, NM1 and GWD2 recorded marked weight losses (- 19.2 mg and—9.3 mg on average for NM1 in experiments one and two and—12.4 mg on average for GWD2) as compared to aphids fed on Védrantais. In contrast, C6 maintained its weight when feeding on [Védrantais]^R_PI161375^ compared to Védrantais melon plants (+ 1.5 mg on average). Interestingly, of all the clones used, C6, which is *Vat*-adapted but did not trigger resistance on [Védrantais]^R_PI161375^ had the lowest standard deviation for weight (on average 0.5 mg on Védrantais and 1.1 mg on [Védrantais]^R_PI161375^), indicating it had the most stable capacity for nutrient acquisition from its source phloem whether feeding on *Vat* or non-*Vat* melon plants. On the contrary, GWD2, which is *Vat*-adapted and which did trigger resistance in [Védrantais]^R_PI161375^, had a marked weight standard deviation, (on average 4.9 mg on Védrantais and 2.5 mg on [Védrantais]^R_PI161375^), indicating a variable capacity to acquire nutrients from either melon genotype. These results indicate that C6 was the most performant clone in terms of weight gain and weight stability on [Védrantais]^R_PI161375^ melon plants.

## Discussion

As a strictly phloemophagous aphid species, *A. gossypii* must deal with an unbalanced diet since phloem sap, containing excess sugars and lacking amino acids, has a high C/N ratio. The cucurbit dual phloem system may offer a possibility for aphids to feed from the EFP which is enriched in nitrogenous compounds and might thereby better fulfil the nutritional needs of aphids than the FP [14, 29, 40], despite the presence of potentially toxic and/or repellent metabolites [18, 20].

### RFO accumulation in resistant plants does not affect aphids, but may play a role in aphid adaptation to fasting

In plants, RFO were suggested to be inducible under biotic stresses [35]. In aphid-infested melon plants, levels of both stachyose and sucrose remained invariant as compared to the control plants, while galactinol, which is a precursor for RFO synthesis, varied significantly between aphid-infested and healthy plants **(**Fig. 3**)**. In [Védrantais]^R_PI161375^, infestation by GWD2 and NM1 led to an accumulation of galactinol while infestation by C6 led to a decrease in galactinol. As both NM1 and GWD2 trigger resistance when feeding on [Védrantais]^R_PI161375^, in contrast to C6, it appears that triggering resistance led to an induction of galactinol synthesis. Galactinol biosynthesis has been shown to increase during biotic stress [21] and evidence suggested it is involved at least in systemic resistance induced by fungal and microbial pathogens [8, 21]. Also, galactinol exhibits antioxidant properties and, in plants challenged by biotic stress where accumulation of reactive oxygen species typically occurs, it may alter such a cell response [35]. In *Vat*-melon plants, an oxidative burst is known to occur upon infestation by *A. gossypii* [37], but this reaction is limited to cells close to the aphid's probing site. Accumulation of galactinol could explain the restricted oxidative burst when resistance was triggered.

For aphids, the deterrent and negative impact of RFOs on the fecundity of *M. persicae* have been reported in choice and non-choice trials using transgenic RFO-producing *Arabidopsis thaliana* plants and on RFO-supplied artificial media (for example: galactinol, sucrose, raffinose, stachyose). Nevertheless, their toxicity to aphids was suggested to be indirect, potentially contributing to other defence responses in plants, such as the activation of secondary metabolism [4, 5]. NM1 accumulated more stachyose, sucrose and galactinol when feeding on Védrantais than on [Védrantais]^R_PI161375^ while it multiplied better on Védrantais, showing that these RFOs had no direct effect on *A. gossypii* performance (Figs. 1 and S2).

Moreover, we observed that stachyose, sucrose and galactinol, but not trehalose or raffinose, were purged from aphids following fasting. Trehalose is known to be a common hemolymph sugar storage form in insects and a possible dehydration tolerance agent [32], suggesting it might never totally disappear from aphids and that its accumulation might be favoured in response to a fasting period when dehydration occurs. Raffinose is the intermediate form between sucrose and stachyose in the RFO biosynthesis pathway [31], but while sucrose and stachyose could be purged, raffinose could not (Fig. 4), suggesting its accumulation could confer a potential advantage to the aphid.

### Cucurbitacin *in planta* is not a real threat to *Aphis gossypii*

LC–MS analysis revealed the presence in leaves of the aglycone cucurbitacin B (Cb) and its glycosylated derivative (Cb-Glc), which has been previously reported in cucurbits [19, 28]. Both forms were detected in highly variable amounts in melon genotypes (Fig. 2), suggesting that genotypes and growing conditions might influence cucurbitacin synthesis in melon.

In general, the glycosylated derivatives of secondary metabolites are inactive storage forms which are easily mobilized in response to attacks by pests [16, 41]; however, neither Cb nor Cb-Glc levels were modified upon infestation, independently of the triggering of *Vat-*mediated resistance (Fig. 3). This was unexpected as cucurbitacins have already been shown to be inducible upon herbivory [18]. In aphid samples, Cb-Glc was not detected, and in *A. gossypii* in particular, the description of a mechanism of glycosyltransferase-mediated detoxification of allelochemicals has already suggested that the glycosylated derivatives are non-toxic and easily excreted by aphids [25]. In contrast, aglycone allelochemical forms, and notably Cb, are well known for their toxic/repellent effect on insects [13, 18, 33, 39]. In *A. gossypii*, such negative impacts of Cb have only been reported in vitro or via the application of exogenous cucurbitacin on melon leaves [39, 42] and interestingly Cb was detected in a few batches of *A. gossypii*, suggesting aphids can accumulate certain amounts of Cb during the experiment, even when they fed on *Vat*-melon. Remarkably, Cb levels were very low in the two *Vat* gene donor lines, PI 161,375 and PI 482,398 (Fig. 2). Altogether, these results suggest that cucurbitacin-related compounds are not involved in melon resistance to *A. gossypii*.

If not a threat in vivo, Cb detoxifying/excreting mechanisms in *A. gossypii* may contribute to its specialization as cucurbit host-race clones. Nevertheless, as Cb was only detected in a few aphid samples and always in low quantities, the hypothesis of an adaptation to cucurbitacin might not be relevant, even for highly adapted clones such as NM1, C6 and GWD2 that could not be multiplied on okra, a Malvaceae species expected to be a generalist host for *A. gossypii* [6].

### *A. gossypii* clones only feed from cucurbit FP even in resistant melon

Current knowledge on aphid interaction with the dual phloem system in cucurbits is quite sparse. Using stylectomy, Kanvil et al. [20] showed *M. persicae* fed exclusively from the FP. However, it is less obvious in the case of A. *gossypii,* as only indirect evidence is available [20]. We showed that all three *A. gossypii* clones exploited the FP as a food source, independently of the plant genotype on which they fed. Remarkably, the two *Vat*-adapted clones, GWD2 and C6, accumulated the same amount of FP markers when fed on Védrantais or [Védrantais]^R_PI161375^, suggesting that both clones accessed the FP, and that when resistance was triggered, *e.g.* by GWD2 on [Védrantais]^R_PI161375^, access to the FP was not impaired. Nevertheless, GWD2 weighed less on [Védrantais]^R_PI161375^ than on Védrantais melon plants, whereas C6 only displayed on average a slightly higher weight on [Védrantais]^R_PI161375^. In light of these results, and bearing in mind that C6 does not trigger resistance in [Védrantais]^R_PI161375^ whereas GWD2 does, it seems that triggering resistance impaired the nutritional quality of melon plants for the clones which are adapted to *Vat*.

Cb was detected in a few batches of *A. gossypii* fed on melon plants, always at trace levels, suggesting that aphids can probe EFP but only occasionally and unsustainably. The weight loss for NM1 feeding on [Védrantais]^R_PI161375^ was thus probably unrelated to EFP feeding, which has been confirmed by electrical penetration graph studies showing that NM1 on *Vat* plants had difficulty probing, and in the rare cases it reached the phloem, it did not sustainably feed [2]. As for GWD2, a clone adapted to *Vat*, sustained feeding from the FP occurred, as revealed by aphid sugar contents, but reaching the phloem could take longer on [Védrantais]^R_PI161375^ than on Védrantais, potentially explaining the aphid weight loss measured over the short duration of the experiment (17 h).

Therefore, we have shown that *A. gossypii* cucurbit host-race clones fed from the FP of susceptible melon and that adapted clones on resistant melon also fed from the FP. To feed successfully from the FP, *A. gossypii* has to overcome sieve plate occlusion which was shown in resistant melon when infested by *A. gossypii* [26]. Components of aphid watery saliva have been suggested to play a role in preventing and/or reversing sieve plate occlusion by proteins, callose plugs and sieve element protein-lining [1, 36]. In the melon-*A. gossypii* interaction system, it has been shown that aphid recognition by *Vat* induced callose and lignin deposition on the cell walls along the stylet path [37]. The decrease in callose deposition has been suggested to involve Ca^2+^ sequestration by aphid salivary effectors [36]. Cucurbit sieve-element proteins PP1 and PP2 were degraded in vitro by the saliva of *Acyrthosiphon pisum* and *Macrosiphum euphorbiae* – both species not found to colonize cucurbits [15]. Therefore, the role of effectors is probably a major part of the ability of aphids to colonize cucurbits, suggesting that evolution of phloem complexity did not play a role in the ability of *A. gossypii* to specialize on cucurbits.

## Materials and methods

### Plant and aphid material

We used two aphid-resistant melon lines: PI 161,375 from Korea, and PI 482,398 from Zimbabwe, and two aphid-susceptible melon lines: Védrantais and Charentais T from France. The melon lines were obtained from the Centre for Vegetable Germplasm (CRB-Leg) [30]. Accessions with their genebank identifier, year of introduction and origin are as follows:

- PI 161,375: ME00591 (1967), Korea
- PI 482,398: ME00483 (1997), Zimbabwe
- Védrantais: ME01000 (1982) a commercial variety originally supplied by Vilmorin
- Charentais T: ME00695 (1980) part of the French national collection, originally supplied by Vilmorin

Seed exchange complied with all obligations arising from, and does not contravene any specific use restrictions of: the United Nations’ Convention on Biological Diversity as entered into force on December 29, 1993 (CBD); the Nagoya Protocol on Access to Genetic Resources and the Fair and Equitable Sharing of Benefits Arising from their Utilization to the Convention on Biological Diversity as entered into force on October 12, 2014; the Convention on International Trade in Endangered Species of Wild Fauna and Flora as entered into on March 3, 1973 (CITES); and all their implementing and other relevant national, local or indigenous access and benefit-sharing laws.

We also used a transgenic Védrantais resistant line (built by the team) with an insertion of an 11-kb genomic DNA sequence which included the *Vat* allele under the control of its own promoter isolated from PI 161375 [12], named [Védrantais]^R_PI161375^. This line was used according to the agreement DUO 7306 (2020–2025) delivered by the French ‘Ministère de l’enseignement supérieur, de la recherche et de l’innovation the 2020/06/22. We also used a near-isogenic line [Charentais T]^R_PI482398^, (built by the team), derived from a cross between PI 482398 and Charentais T, followed by six backcrosses with the Charentais T parental line. At each generation, plants showing resistance to NM1 were selected. No voucher specimen of this material has been deposited in a publicly available herbarium. Plantlets were grown in an insect-proof greenhouse up to the two-leaf stage and were subsequently used for biological tests.

We used three *A. gossypii* clones of the cucurbit host-race, which produced contrasted patterns of resistance on *Vat* melon plants, *i.e.* plants carrying a resistant allele in the *Vat* gene cluster. Clones were maintained by synchronous mass rearing on Védrantais melon, or on okra, a plant from the Malvaceae family expected to act as a host for any aphid host race [6] at 24 °C/18 °C under a 16 h/8 h day/night photoperiod.

### Experimental design and time course for plant/aphid interaction study

#### Experimental design

Two independent experiments were conducted. Experiment 1 used NM1 and GWD2 and experiment 2 used NM1 and C6. Both experiments used the Védrantais and [Védrantais]^R_PI161375^ melon plants. Plants and aphids were placed in a growth chamber at 24/18 °C (day/night), with a photoperiod of 16/8 h (day/night). Védrantais and [Védrantais]^R_PI161375^ plants were arranged in staggered rows to reduce positional effects. Plantlets were grown in trays filled with water, each tray containing 6 plantlets with sufficient space between plantlets to avoid leaf overlap. Plastic rings were glued to the base of each of the two plantlet limbs in order to prevent aphids escaping from the leaves (Fig. S3).

#### Experimental time course

At time zero (t0), batches of 60 mass-reared aphids were transferred to small glass petri dishes using a moist paintbrush, which were then sealed using parafilm. Aphids were then fasted in vitro for 4 h. After four hours (t = 4 h), 5 batches of NM1 and 9 batches of GWD2 (experiment 1) and 3 batches of NM1 and 7 batches of C6 (experiment 2; used as control for fasting efficiency), were transferred to 2 mL Eppendorf tubes. The samples were weighed, then immediately frozen and stored at -80 °C.

At t = 4 h, the other batches were gently deposited onto a plantlet (60 aphids per plants *i.e.* 30 aphids per limb) with a paintbrush. For the first experiment, batches of NM1 and GWD2 were deposited onto nine Védrantais and [Védrantais]^R_PI161375^ plants, for the second experiment, batches of NM1 and C6 were deposited onto seven plants of each genotype. The same number of control plantlets underwent the same treatments as the aphid-infested ones: with plastic rings glued at the base of their two leafstalks and using a paintbrush to simulate aphid deposition. After 21 h (t = 21 h), aphids were removed from the leaflets and transferred to 2 mL Eppendorf tubes. Pairs of limbs from each plantlet were transferred to 15 mL Corning tubes. Samples were immediately frozen and then stored at—80 °C. A total of 54 plants and 50 aphid batches were sampled during the first experiment and 42 plants and 38 aphid batches were sampled during the second experiment.

### Plant and aphid metabolite extraction

All solvents were analytical or LC/MS grade. Pure standards were purchased from Extrasynthèse (Genay, France) and ChemFaces (Wuhan, China).

Leaf samples were ground into a fine powder in liquid nitrogen, approximately 200 mg of powder were transferred into tubes of 2 ml and then 1.6 ml of methanol acidified with formic acid (0.125%; *v/v*) were added. The tubes were incubated at 70 °C for 10 min in a water bath, 0.4 ml of ultra-pure water were added and the samples were incubated under the same conditions. At each step, the samples were homogenized with a vortex. The samples were centrifuged at 5300 rcf for 5 min at 4 °C and supernatants were filtered through a 0.2 µm membrane. For LC analysis, the aliquots of filtered extracts were stored at—20 °C. For GC analysis, 10 µl of the filtered extracts were dried overnight under vacuum and stored at—20 °C.

Aphid samples were ground directly in the extraction solvent: 0.8 ml of methanol acidified with formic acid (0.125%; *v/v*) were added and the samples were ground in a mixer mill containing two cooled steel balls for 45 s with 30 oscillations per second. The samples were incubated in a water bath at 70 °C for 10 min and homogenized twice with a vortex during the incubation period. Then, 0.2 ml of ultrapure water were added and the tubes were placed back in the water bath. The extracts were filtered through a 0.2 µm membrane.

For LC analysis, 700 µl of the filtered extracts were concentrated to dryness in a vacuum concentrator and stored at -20 °C. Immediately before injection, the dried residue was solubilized with 175 µl of methanol. For GC analysis, 50 µl of the filtered extracts were dried under vacuum and stored at—20 °C.

### Liquid Chromatography-Mass Spectometry (LC–MS)

Ultra-Performance Liquid Chromatography (UPLC) was carried out using an Acquity UPLC Class I (Waters, Milford, MA, USA). Metabolite separation was carried out on a reverse phase column, BEH C18 1.7 µm 2.1*100 mm, equipped with a guard column (Waters, USA). The mobile phase, consisting of water with formic acid (0.1%, v/v) (eluent A) and acetonitrile with formic acid (0.1%, v/v) (eluent B), was pumped at 0.4 ml/min. The eluent gradient was: 0–0.5 min: 2% B; 0.5–6.5 min: 2–44.5% B; 6.5–7.5 min: 44.5–100% B; 7.5–9 min: 100% B. Then, the concentration of B was decreased to 2% and the column was equilibrated before the next injection (total run time 10 min). The column temperature was held at 35 °C and the samples at 10 °C. For leaf samples, 1 µL was injected. For aphid samples, 4 µL were injected. The UPLC system was coupled with a triple quadripole mass spectrophotometer TQ-XS XEVO (Waters, Milford, MA, USA) with an electrospray source at 150 °C in positive ion mode with a 3.5 kV capillary voltage, 550 °C desolvation temperature, 1000 L/h desolvation gas flow and 150 L/h scanning cone flow. The system was controlled by Masslynx version 1.2 (Waters, Milford, MA, USA).

**Fig. 7:**
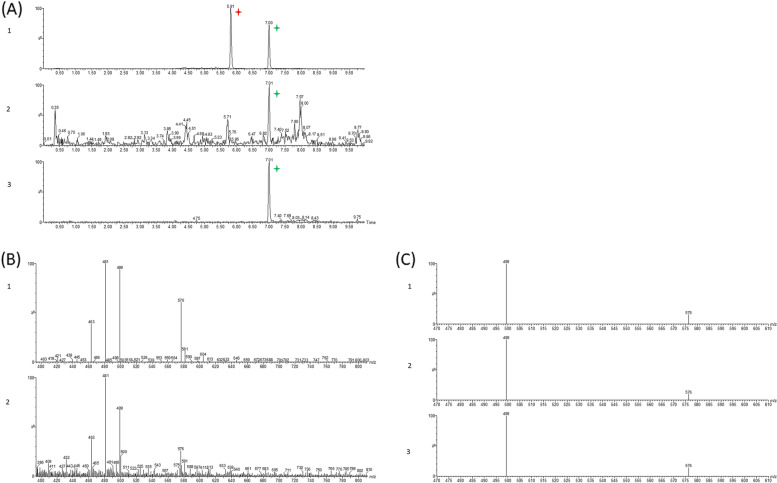
Identification by LC–MS of cucurbitacin B and glycosylated cucurbitacin B in aphids and melons.** A** LC–MS profiles of cucurbitacins (Extracted Ion Chromatogram of ion 499.2). 1: leaf extract, 2: aphid extract, 3: authentic standard of cucurbitacin B. Peak height was normalised to that of the largest peak. Red mark: glycosylated cucurbitacin B, green mark: cucurbitacin B. **B** LC–MS mass spectra of: 1: cucurbitacin B standard, 2: leaf extract at t_R_ = 7.01 min. The peak heights were normalised to that of the largest peak. **C** MRM spectra of the two transitions: 576.3 > 499.2 and 499.2 > 481.2 at t_R_ 7.01 min, showing the ratios between the two ions produced, 576 and 499: 1: cucurbitacin B standard (ratio 5.6), 2: aphid extract (ratio 6.7), 3: leaf extract (ratio 6.3)

To optimize the detection of cucurbitacins, we injected authentic standards of cucurbitacin B (Cb) and cucurbitacin B 2-O-beta-D-glucoside (Cb-Glc) (Fig. 7A).

In scan mode (100–1000 uma), mass spectra of Cb revealed, at the retention time (t_R_) of 7.01 min, the ammonium adduct [M + NH_4_]^+^ (m/z 576.3) and a direct fragmentation into ions 499, 481 and 463, which correspond to losses of the methyl ester group, and two water molecules (Fig. 7B). Mass spectra of Cb-Glc revealed, at a t_R_ of 5.3 min, the ammonium adduct [M + NH_4_]^+^ (m/z 738,0) as well as fragments of the 499, 481 and 463 ions. We developed a multiple reaction monitoring (MRM) method using two transitions 576.3 > 499.2 and 499.2 > 481.2, with a cone voltage of 20 V and a collision energy of 60 V. We used the ion abundance measurement of m/z 499.2 for quantification and made an external calibration using a range of cucurbitacin concentrations.

In order to ensure the detection of Cb and Cb-Glc in aphids, we measured the abundance of ions of m/z 499 and 576; we then calculated the ratio of areas measured and compared this with the ratios obtained for the standards (Fig. 7C). The limit of quantification in aphids was 0.3 nM in extracts and 0.005 nM.mg^1^.

### Gas Chromatography – Mass Spectrometry (GC–MS)

Sample derivatization was performed online using a multipurpose sampler (Gerstel MPS, CTC Analytics AG, Mülheim an der Ruhr, Switzerland). The dry extracts were incubated in 50 µl of a pyridine solution containing 20 mg/ml of methoxyamide hydrochloride. Incubation was carried out under shaking at 250 rpm for 6 min at 80 °C, followed by 12 min at room temperature. Subsequently, 80 μl of BSTFA (N,O-Bis(trimethylsilyl)trifluoroacetamide) containing a mixture of 9 n-alkanes were added and the total mixture was heated for 30 min with constant stirring at 250 rpm. Derivatization was then continued for 52 h at room temperature. The separation of the metabolites of the derived samples was carried out with a gas chromatography system (7890B GC, Agilent Technologies, Santa Clara, CA, USA) equipped with a capillary column (ZB-SemiVolatiles, 34.59 m, internal diameter 250 µm, film thickness 250 µm, Phenomenex, Torrance, USA) coupled to a time-of-flight (TOF) mass spectrometer (Pegasus BT, Leco, Saint Joseph, Benton Harbor, MI, USA). One microlitre of sample was injected in splitless mode at 270 °C with helium as the carrier gas at 0.6 ml/min. The oven temperature was held at 80 °C for 1 min, then gradually increased to 330 °C (18 °C/min) and held for 15 min after the set temperature was reached. Source and transfer line temperatures were set at 250 °C. The acquisition was run after a delay of 700 s. Mass acquisitions were performed in scan mode in the m/z range of 50–630 with a cycle time of 10 scans/s. The multipurpose sampler was controlled by Maestro Version 1.4.40.1. The gas chromatography system and mass spectrometer were controlled by ChromatTOF Version 5.20.38.0.54864 (LECO, Saint Joseph, MI, USA). Data were deconvoluted using the LECO NTD software (LECO, USA), and the annotation was confirmed with the injection of pure standards. The amount of compounds was calculated as the area under the GC–MS peak representing each compound analysed, normalized by the weight of the corresponding sample (mg).

**Fig. 8:**
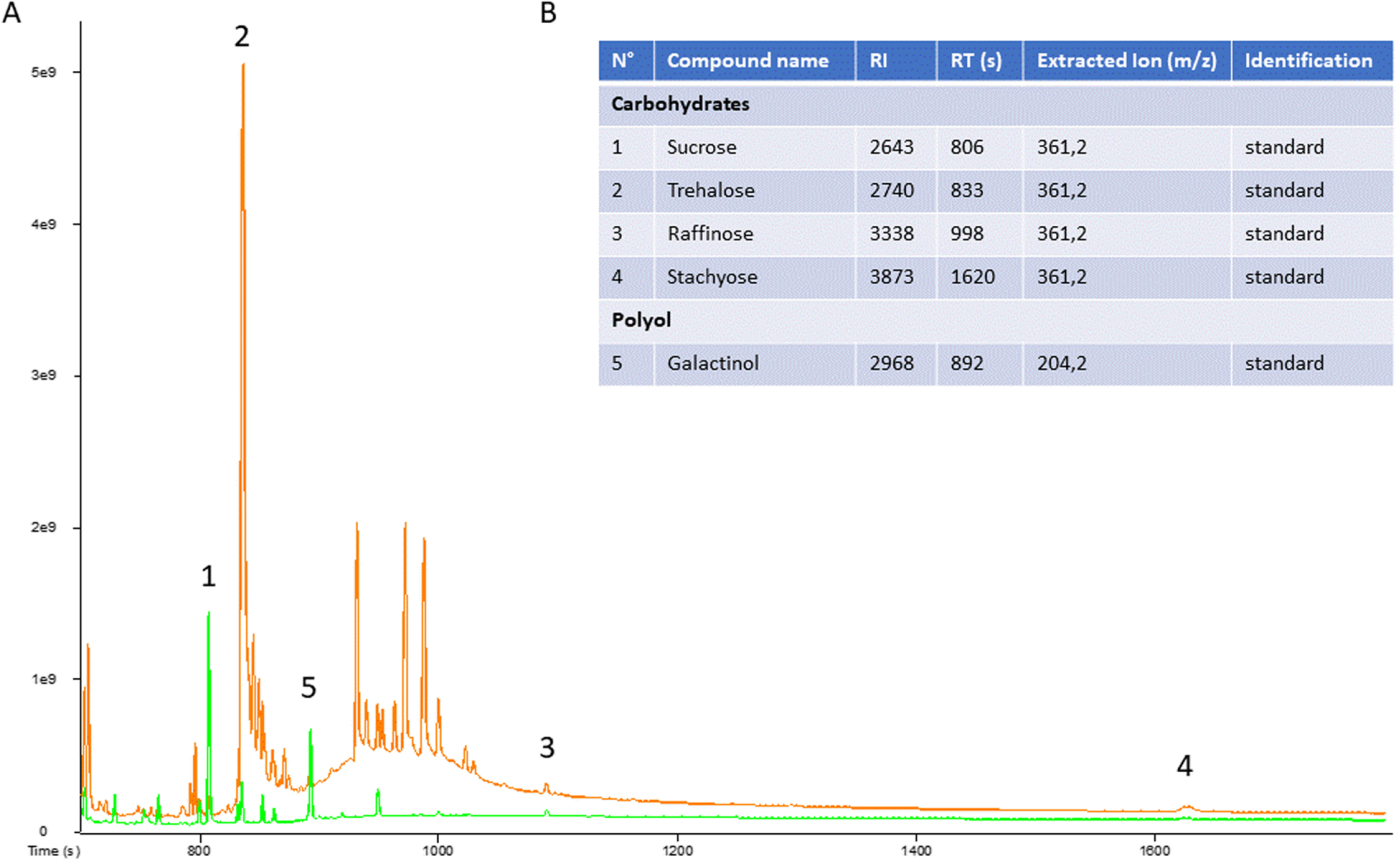
Identification by GC–MS of four sugars and galactinol in aphids and melons.** A** GC–MS profiles of extracts of leaves (green) and aphids (orange). Total Ion Current chromatogram showing 1: Sucrose, 2: Trehalose, 3: Raffinose, 4: Stachyose, 5: Galactinol. **B** list of compounds identified by GC–MS. RI: retention index, RT: retention time

Standard solutions of sucrose, trehalose, raffinose, stachyose and galactinol were injected. Two extracted ion chromatograms (XIC) were selected for signal measurement. We used the sugar-typical XIC of m/z 361.2 for sucrose, trehalose, raffinose and stachyose and used the XIC of m/z 204.2 for galactinol (Fig. 8).

### Chromatogram analysis

Integration of chromatography peaks to infer quantities of metabolites was carried out with TargetLynx (Waters, USA). No quantifiable traces were reported as ‘null’. Both Cb and Cb-Glc were quantified using a calibration curve of authentic standards of Cb, Cb-Glc being expressed as equivalent Cb. For metabolites analysed by GC–MS, the areas under the peaks of a characteristic extracted ion chromatogram (EIC) were used for relative quantification. All statistical analyses were conducted with XLSTAT software (AddinSoft, Paris, France). Kruskal–Wallis tests were performed using a Monte Carlo method.

## Supplementary Information

**Additional file 1: Table S1.** Quantification of seven phloem-associated metabolites in six melon lines. Stachyose, trehalose, sucrose, raffinose and galactinol were analysed by GC-MS and quantified as the area under the chromatogram peak (no units) corrected by leaf sample weight (g). Cucurbitacin B and Glycosylated Cucurbitacin B were analysed by LC-MS and expressed as nM.mg^-1^. PI 161375 and PI 482398 melons carry different *Vat* resistance alleles, Védrantais and Charentais T are susceptible to aphids. Their isogenic and near-isogenic lines with resistance donors are noted [Védrantais]^R_PI 161375^ and [Charentais]^R_PI 482398^. **Table S2.** Quantification of five phloem-associated metabolites in healthy versus aphid-infested melon plants. Védrantais and its isogenic [Védrantais]R_^PI 161375^ plants were infested for 17h by NM1 aphid clone -triggering the *Vat*-mediated resistance- in both experiments, in experiment (1) by GWD2 aphids -triggering too the *Vat*-mediated resistance- and in experiment (2) by C6 aphids -no triggering the *Vat*-mediated resistance. Stachyose, sucrose and galactinol were analysed by GC-MS in melon leaves and expressed as peak area (no units) corrected by leaf sample weight (g). Cucurbitacin B and Glycosylated Cucurbitacin B were analysed by LC-MS and expressed as nMole.mg^-1^. **Table S3.** Quantification of seven metabolites in Okra and Védrantais melon plants. Sucrose, trehalose, galactinol, raffinose and stachyose were analysed by GC-MS and expressed as peak area/mg. Cucurbitacin B (Cb) and Glycosylated Cucurbitacin B (Cb-Glc) were analysed by LC-MS and expressed as nM/g. **Table S4.** Seven melon-associated metabolites in aphids during fasting. C6 adult aphids were reared on Védrantais melon plants, then fasted by batches of 20 to 50 individuals for 3h, 6h and 23h. t0 corresponds to the aphids sampled at rearing (before fasting). Stachyose, sucrose and galactinol were analysed by GC-MS in aphid batches and expressed as peak area (no units) corrected by the aphid batch weight (mg). Cucurbitacin B and Glycosylated Cucurbitacin B were not detected by LC-MS. **Table S5.** Stachyose, sucrose and galactinol in three aphid clones fed on Vat/non-Vat melons. Aphid clones were fed on Védrantais and its isogenic [Védrantais]^R_PI161375^ melon lines for 17h. NM1 aphids -triggering the *Vat*-mediated resistance- were used in both experiments, with either GWD2 aphids -triggering the resistance in [Védrantais]^R_PI161375^- in experiment (1), or C6 aphids -non-triggering the resistance in [Védrantais]^R_PI161375^- in experiment (2). Results are ratios of [metabolite quantity in an aphid batch]/[metabolite quantity in the leaf on which they fed]. **Table S6.** Quantification of stachyose, sucrose and galactinol in three aphid clones fed on Vat/non-Vat melon. Aphid clones were fasted for 4h and then fed on Védrantais and its isogenic [Védrantais]^R_PI161375^ melon lines for 17h. NM1 aphids -triggering the *Vat*-mediated resistance- were used in both experiments, with either GWD2 aphids -triggering the resistance in [Védrantais]^R_PI161375^- in experiment (1), or C6 aphids -non-triggering the resistance in [Védrantais]^R_PI161375^- in experiment (2). Stachyose, sucrose and galactinol were analysed by GC-MS in aphid batches and expressed as peak area (no units) corrected by the batch weight (mg). **Table S7.** Cucurbitacin B and Glycosylated Cucurbitacin B in aphids fasted and fed *on Vat/non-Vat *melons. Aphids were fasted for 4h and then fed on Védrantais and its isogenic [Védrantais]^R_PI161375^ melon lines for 17h. NM1 aphids -triggering the *Vat*-mediated resistance and GWD2 aphids -triggering the resistance in [Védrantais]^R_PI161375^. Results are expressed as peak area (no units) corrected by the aphid batch weight (mg). **Table S8.** Weight (mg) of three aphid clones fasted and fed on Vat/non-Vat melons. Aphids were fasted for 4h and then fed on Védrantais and its isogenic [Védrantais]^R_PI161375^ melon lines for 17h. NM1 aphids -triggering the *Vat*-mediated resistance- were used in both experiments, with either GWD2 aphids -triggering the resistance in [Védrantais]^R_PI161375^- in experiment (1), or C6 aphids -non-triggering the resistance in [Védrantais]^R_PI161375^- in experiment (2).

**Additional file 2: Fig. S1.** Quantification of seven metabolites in Okra and Védrantais melon plants. In Okra (blue boxplots) and in the Védrantais melon line (green boxplots). Sucrose, trehalose, galactinol, raffinose and stachyose were analysed by GC-MS and expressed as peak area/mg. Cucurbitacin B (Cb) and Glycosylated Cucurbitacin B (Cb-Glc) were analysed by LC-MS and expressed as nM/g. The number of observations per plant genotype was 5. **Fig. S2.** Quantification of stachyose, sucrose and galactinol in three aphid clones fasted or fed on Vat/non-Vat melons. Aphid clones were fasted for 4h and then fed on Védrantais and its isogenic [Védrantais]^R_PI161375^ melon lines for 17h. NM1 aphids -triggering the Vat-mediated resistance- were used in both experiments, with either GWD2 aphids -triggering the resistance in [Védrantais]^R_PI161375^- in experiment (1), or C6 aphids -non-triggering the resistance in [Védrantais]^R_PI161375^- in experiment (2). Stachyose, sucrose and galactinol were analysed by GC-MS in aphid batches and expressed as peak area (no units) corrected by the batch weight (mg). The number of observations is between 3 and 9. Letter codes indicate significant differences between fed aphids at level alpha=0.05 by Kruskal-Wallis analysis. **Fig. S3.** Growth chamber with the experimental design for aphids feeding melon plants. (A): Global design. Each side was used for a different clone. Beige coloured plant pots corresponded to Védrantais and brown plant pots to [Védrantais]^R^^_PI161375^. The picture was taken one day before aphid infestation, during plant acclimation to the growth chamber. (B): 15-day old melon plantlets infested with a batch of aphids (C6). Red plastic rings were glued at the base of each of the infested limbs to prevent aphids escaping from leaves.

## Data Availability

All data are available in a supplementary file and in the Data Gouv repository accessible at https://doi.org/10.57745/3LI3FJ.

## References

[1] Åhman I, Kim SY, Zhu LH (2019). Plant Genes Benefitting Aphids—Potential for Exploitation in Resistance Breeding. Front Plant Sci.

[2] Boissot N, Schoeny A, Vanlerberghe-Masutti F (2016). *Vat*, an amazing gene conferring resistance to aphids and viruses they carry: From molecular structure to field effects. Front Plant Sci.

[3] Boissot N, Thomas S, Chovelon V, Lecoq H (2016). NBS-LRR-mediated resistance triggered by aphids: Viruses do not adapt; aphids adapt via different mechanisms. BMC Plant Biol.

[4] Cao T. Metabolic engineering of raffinose-family oligosaccharides in the phloem reveals alterations in patterns of carbon partitioning and enhances resistance to green peach aphid. University of North Texas. 2010. 10.3389/fpls.2013.00263 PMC3715723 23882277

[5] Cao T, Lahiri I, Singh V, Louis J, Shah J, Ayre BG Metabolic engineering of raffinose-family oligosaccharides in the phloem reveals alterations in carbon partitioning and enhances resistance to green peach aphid. Front Plant Sci. 2013;4:1–13. 10.3389/fpls.2013.00263. 10.3389/fpls.2013.00263 PMC3715723 23882277

[6] Carletto J, Lombaert E, Chavigny P, Brévault T, Lapchin L, Vanlerberghe-Masutti F (2009). Ecological specialization of the aphid *Aphis gossypii* Glover on cultivated host plants. Mol Ecol.

[7] Chen JQ, Martin B, Rahbé Y, Fereres A (1997). Early intracellular punctures by two aphid species on near-isogenic melon lines with and without the virus aphid transmission (*Vat*) resistance gene. Eur J Plant Pathol.

[8] Cho SM, Kang EY, Kim MS, Yoo SJ, Im YJ, Kim YC, Yang KY, Kim KY, Kim KS, Choi YS, Cho BH (2010). Jasmonate-dependent expression of a galactinol synthase gene is involved in priming of systemic fungal resistance in Arabidopsis thaliana. Botany.

[9] Chovelon V, Feriche-Linares R, Barreau G, Chadoeuf J, Callot C, Gautier V, Le Paslier MC, Berad A, Faivre-Rampant P, Lagnel J, Boissot N (2021). Building a cluster of NLR genes conferring resistance to pests and pathogens: the story of the *Vat* gene cluster in cucurbits. Horticulture Research.

[10] Crafts AS (1932). Phloem anatomy, exudation, and transport of organic nutrients in curcurbits. Plant Physiol.

[11] Dangl JL, Horvath DM, Staskawicz BJ (2013). Pivoting the plant immune system from dissection to deployment. Science.

[12] Dogimont C, Chovelon V, Pauquet J, Boualem A, Bendahmane A. The *Vat* locus encodes for a CC-NBS-LRR protein that confers resistance to *Aphis gossypii* infestation and *A. gossypii*-mediated virus resistance. Plant J. 2014;80(6):993–1004. 10.1111/tpj.12690. 10.1111/tpj.12690 25283874

[13] Elsayed G (2011). Plant secondary substances and insects behaviour. Arch Phytopathol Plant Protect.

[14] Fiehn O (2003). Metabolic networks of *Cucurbita maxima* phloem. Phytochemistry.

[15] Furch ACU, Van Bel AJE, Will T (2015). Aphid salivary proteases are capable of degrading sieve-tube proteins. J Exp Bot.

[16] Gachon CMM, Langlois-Meurinne M, Saindrenan P (2005). Plant secondary metabolism glycosyltransferases: The emerging functional analysis. Trends Plant Sci.

[17] Garzo E, Fernández-Pascual M, Morcillo C, Fereres A, Gómez-Guillamón ML, Tjallingii WF (2018). Ultrastructure of compatible and incompatible interactions in phloem sieve elements during the stylet penetration by cotton aphids in melon. Insect Science.

[18] Gaupels F, Ghirardo A. The extrafascicular phloem is made for fighting. Front Plant Sci. 2013;4:2–5. 10.3389/fpls.2013.00187. 10.3389/fpls.2013.00187 PMC3678090 23781225

[19] Haq FU, Ali A, Khan MN, Shah SMZ, Kandel RC, Aziz N, Adhikari A, Choudhary MI, Ur-Rahman A, El-Seedi HR, Musharraf SG (2019). Metabolite Profiling and Quantitation of Cucurbitacins in Cucurbitaceae Plants by Liquid Chromatography coupled to Tandem Mass Spectrometry. Sci Rep.

[20] Kanvil S, Pham J, Lopez-Cobollo R, Selby M, Bennett M, Beckingham C, Powell G, Turnbull C (2017). Cucurbit extrafascicular phloem has strong negative impacts on aphids and is not a preferred feeding site. Plant Cell Environ.

[21] Kim MS, Cho SM, Kang EY, Im YJ, Hwangbo H, Kim YC, Ryu CM, Yang KY, Chung GC, Cho BH (2008). Galactinol is a signaling component of the induced systemic resistance caused by *Pseudomonas chlororaphis* O6 root colonization. Mol Plant Microbe Interact.

[22] Klingler J, Powell G, Thompson GA, Isaacs R (1997). Phloem specific aphid resistance in *Cucumis melo* line AR 5: effects on feeding behaviour and performance of *Aphis gossypii*. Entomol Exp Appl.

[23] Mattson WJ (1980). Herbivory in Relation to Plant Nitrogen Content. Annu Rev Ecol Syst.

[24] Mistral P, Vanlerberghe-Masutti F, Elbelt S, Boissot N. Aphis gossypii/Aphis frangulae collected worldwide: Microsatellite markers data and genetic cluster assignment. Data Brief. 2021;36:106967. 10.1016/j.dib.2021.106967. 10.1016/j.dib.2021.106967 PMC8026900 33855139

[25] Nagare M, Ayachit M, Agnihotri A, Schwab W, Joshi R (2021). Glycosyltransferases: the multifaceted enzymatic regulator in insects. Insect Mol Biol.

[26] Peng HC, Walker GP (2018). Sieve element occlusion provides resistance against *Aphis gossypii* in TGR-1551 melons. Insect Science.

[27] Powell G, Tosh CR, Hardie J (2006). HOST PLANT SELECTION BY APHIDS: Behavioral, Evolutionary, and Applied Perspectives. Annu Rev Entomol.

[28] Rajasree RS, Sibi PI, Francis F, William H (2016). Phytochemicals of cucurbitaceae family – A review. Int J Pharmacognosy Phytochem Res.

[29] Richardson PT, Baker DA, Ho LC (1982). The chemical composition of cucurbit vascular exudates. J Exp Bot.

[30] Salinier J, Lefebvre V, Besombes D, Burck H, Causse M, Daunay MC, Dogimont C, Goussopoulos J, Gros C, Maisonneuve B, McLeod L, Tobal F, Stevens R (2022). The INRAE Centre for Vegetable Germplasm: Geographically and Phenotypically Diverse Collections and Their Use in Genetics and Plant Breeding. Plants.

[31] Sengupta S, Mukherjee S, Basak P, Majumder AL. Significance of galactinol and raffinose family oligosaccharide synthesis in plants. Front Plant Sci. 2015;6:1–11. 10.3389/fpls.2015.00656. 10.3389/fpls.2015.00656 PMC4549555 26379684

[32] Thompson SN. Trehalose - The Insect “Blood” Sugar. In Advances in Insect Physiology (Vol. 31, Issue 03). 2003. 10.1016/S0065-2806(03)31004-5.

[33] Torkey HM, Abou-Yousef HM, Azeiz A, Farid HEA (2009). Insecticidal effect of Cucurbitacin E Glycoside isolated from *Citrullus colocynthis* against *Aphis craccivora*. Aust J Basic Appl Sci.

[34] Turgeon R. Phloem Biology of the Cucurbitaceae. Plant Genetics Genomics Crops Models. 2016;291–311. 10.1007/7397.

[35] Valluru R, Van den Ende W (2011). Myo-inositol and beyond - Emerging networks under stress. Plant Sci.

[36] van Bel AJE, Will T. Functional evaluation of proteins in watery and gel saliva of aphids. Front Plant Sci. 2016;7:1–19. 10.3389/fpls.2016.01840. 10.3389/fpls.2016.01840 PMC5156713 28018380

[37] Villada ES, González EG, López-Sesé AI, Castiel AF, Gómez-Guillamón ML (2009). Hypersensitive response to *Aphis gossypii* Glover in melon genotypes carrying the *Vat* gene. J Exp Bot.

[38] Walker GP. Sieve element occlusion: Interactions with phloem sap-feeding insects. A review. J Plant Physiol. 2021;269:153582. 10.1016/j.jplph.2021.153582. 10.1016/j.jplph.2021.153582 34953413

[39] Yousaf HK, Shan T, Chen X, Ma K, Shi X, Desneux N, Biondi A, Gao X (2018). Impact of the secondary plant metabolite Cucurbitacin B on the demographical traits of the melon aphid. Aphis Gossypii Scientific Reports.

[40] Zhang B, Tolstikov V, Turnbull C, Hicks LM, Fiehn O (2010). Divergent metabolome and proteome suggest functional independence of dual phloem transport systems in cucurbits. Proc Natl Acad Sci USA.

[41] Zhang W, Wang S, Yang J, Kang C, Huang L, Guo L. Glycosylation of plant secondary metabolites: Regulating from chaos to harmony. Environment Exp Botany. 2022;194(October 2021):104703. 10.1016/j.envexpbot.2021.104703.

[42] Zhao C, Ma C, Luo J, Niu L, Hua H, Zhang S, Cui J (2021). Potential of cucurbitacin b and epigallocatechin gallate as biopesticides against *Aphis gossypii*. Insects.

