# Supplementary material for: Aphid adaptation to cucurbits: sugars, cucurbitacin and phloem structure in resistant and susceptible melons
Source: BMC Plant Biol. 2023 May 5;23:239. doi: 10.1186/s12870-023-04248-1 (PMC10161555; doi:10.1186/s12870-023-04248-1)
Supplement: Supplementary file 2 — Additional file 2: Fig. S1. Quantification of seven metabolites in Okra and Védrantais melon plants. In Okra (blue boxplots) and in the Védrantais melon line (green boxplots). Sucrose, trehalose, galactinol, raffinose and stachyose were analysed by GC-MS and expressed as peak area/mg. Cucurbitacin B (Cb) and Glycosylated Cucurbitacin B (Cb-Glc) were analysed by LC-MS and expressed as nM/g. The number of observations per plant genotype was 5. Fig. S2. Quantification of stachyose, sucrose and galactinol in three aphid clones fasted or fed on Vat/non-Vat melons. Aphid clones were fasted for 4h and then fed on Védrantais and its isogenic [Védrantais]R_PI161375 melon lines for 17h. NM1 aphids -triggering the Vat-mediated resistance- were used in both experiments, with either GWD2 aphids -triggering the resistance in [Védrantais]R_PI161375- in experiment (1), or C6 aphids -non-triggering the resistance in [Védrantais]R_PI161375- in experiment (2). Stachyose, sucrose and galactinol were analysed by GC-MS in aphid batches and expressed as peak area (no units) corrected by the batch weight (mg). The number of observations is between 3 and 9. Letter codes indicate significant differences between fed aphids at level alpha=0.05 by Kruskal-Wallis analysis. Fig. S3. Growth chamber with the experimental design for aphids feeding melon plants. (A): Global design. Each side was used for a different clone. Beige coloured plant pots corresponded to Védrantais and brown plant pots to [Védrantais]R_PI161375. The picture was taken one day before aphid infestation, during plant acclimation to the growth chamber. (B): 15-day old melon plantlets infested with a batch of aphids (C6). Red plastic rings were glued at the base of each of the infested limbs to prevent aphids escaping from leaves. [file 12870_2023_4248_MOESM2_ESM.pdf]

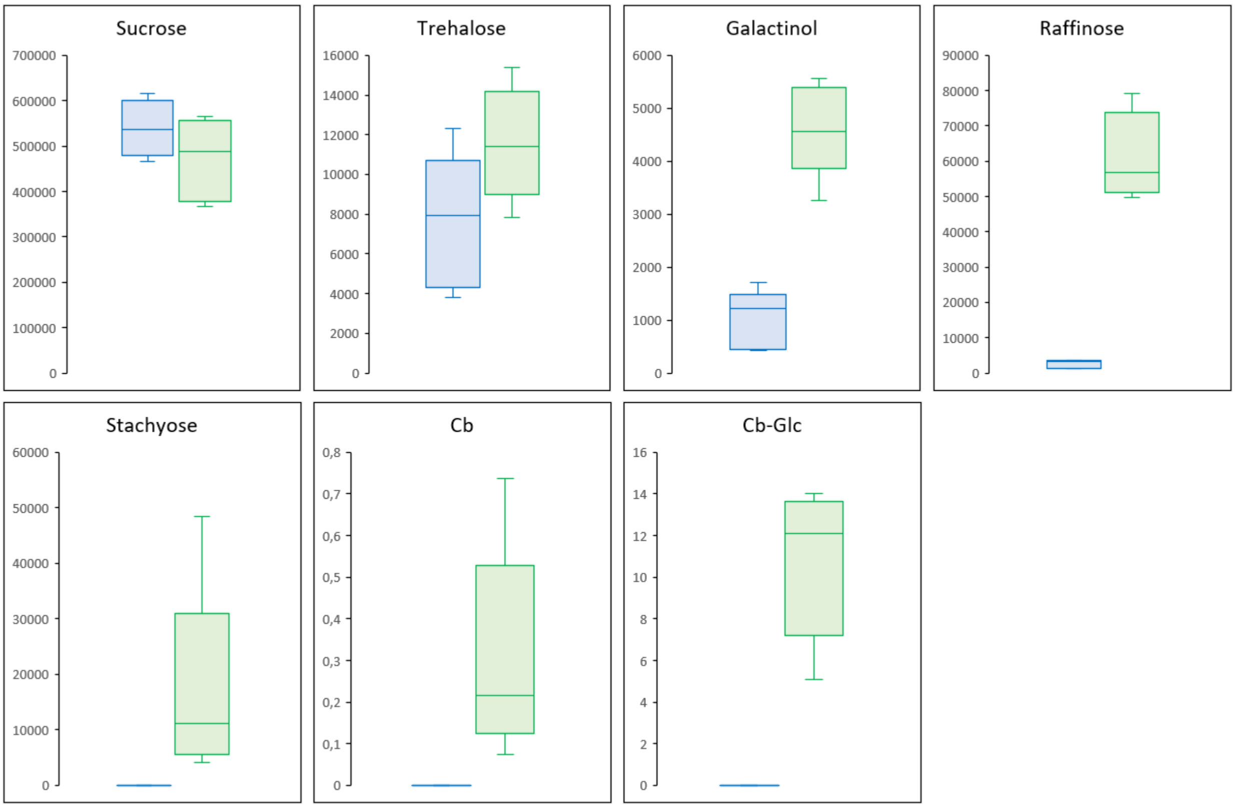

Fig. S 1 – Quantification of seven metabolites in Okra and Védrantais melon plants

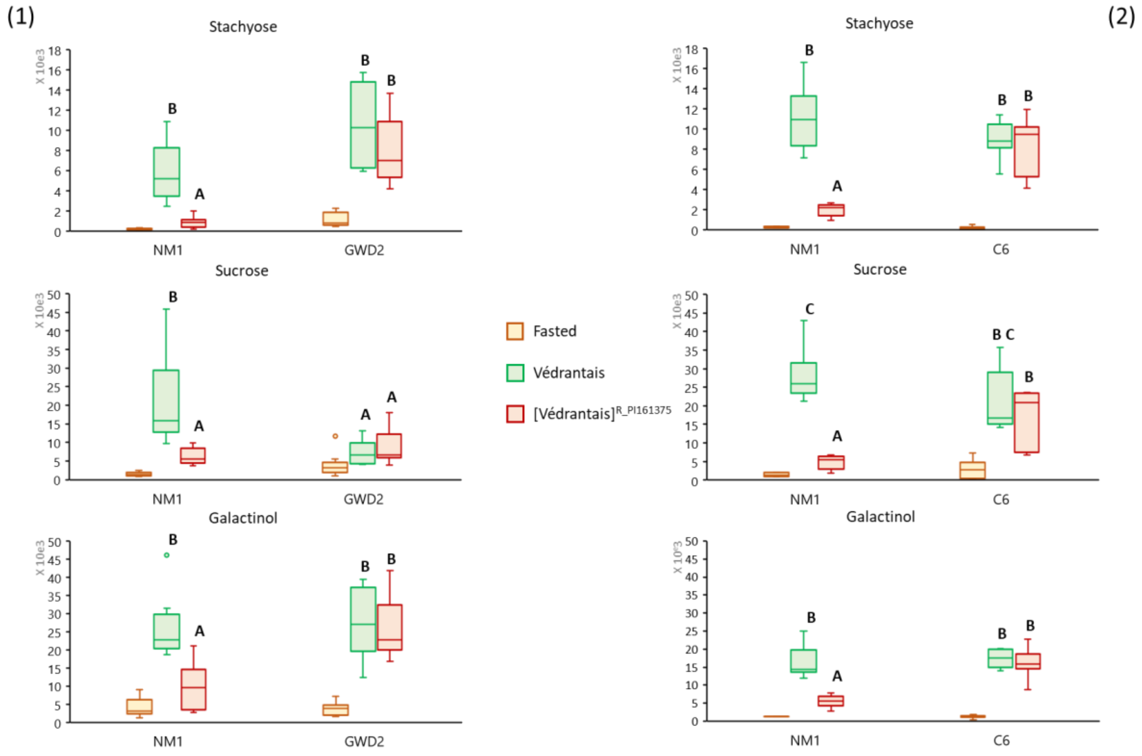

Fig. S 2 – Quantification of stachyose, sucrose and galactinol in three aphid clones fasted or fed on *Vat*/non-*Vat* melons.

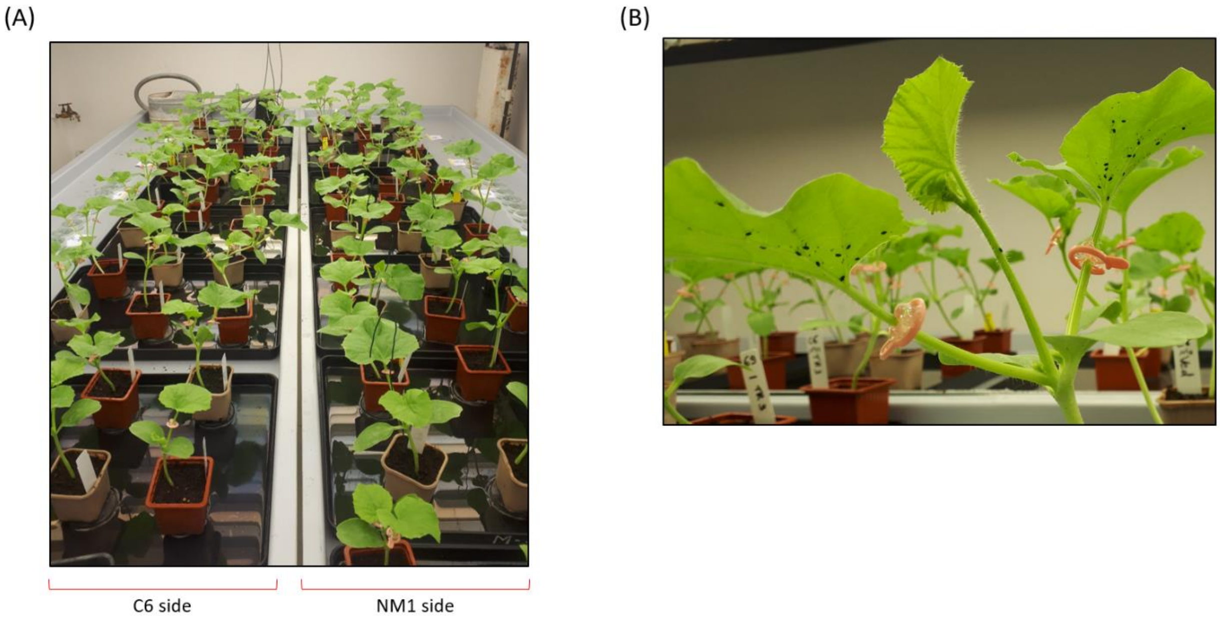

Fig. S 3 – Growth chamber with the experimental design for aphids feeding melon plants
